# Structure Characterization of Polysaccharide from Chinese Yam (*Dioscorea opposite* Thunb.) and Its Growth-Promoting Effects on *Streptococcus thermophilus*

**DOI:** 10.3390/foods10112698

**Published:** 2021-11-04

**Authors:** Jia Ouyang, Feng Wang, Wenjia Li, Qingming Li, Xiaojun Su

**Affiliations:** 1 College of Food Science and Technology, Hunan Agricultural University, Changsha 410128, China; absinthe7@126.com (J.O.); wanglaofeng@163.com (F.W.); liwenjia9301@163.com (W.L.); lqm@hunau.edu.cn (Q.L.); 2 Hunan Provincial Research Center of Engineering and Technology for Fermented Food, Changsha 410128, China

**Keywords:** Chinese yam polysaccharide, structure characterization, *S. thermophilus*, growth-promoting effects

## Abstract

To clarify the mechanisms underlying the growth-promoting effects of yam polysaccharide on *Streptococcus thermophilus (S. thermophilus)*, the yam polysaccharide was extracted using a deep eutectic solvents (DESs) method and separated into four fractions by DEAE-cellulose 52. These fractions were used as the alternative carbon source to substitute lactose to compare their growth-promoting effects on *S. thermophilus*. Furthermore, their molecular weight, monosaccharide and functional groups’ composition, microscopic forms and other basic structure characterizations were analyzed. The results showed that all the fractions could significantly promote *S. thermophilus* growth, and fractions exhibited significantly different growth-promoting effects, whose viable count increased by 6.14, 6.03, 11.48 and 11.29%, respectively, relative to those in the M17 broth medium. Structure-activity relationship analysis revealed that the high growth-promoting activity of yam polysaccharide might be more dependent on the higher molecular weight, the higher galacturonic acid content and its complex spatial configuration, and the existence of β-glycosides would make the yam polysaccharide have a better growth-promoting effect on *S. thermophilus*.

## 1. Introduction

Chinese yam (*Dioscorea opposite* Thunb.) is the tuber of the perennial plant *Dioscorea* (family: *Dioscoreaceae*, *Dioscorea*) [1]. As a medicinal and edible homologous plant, yam has had an edible history for thousands of years. Traditional Chinese medicine believes it has multiple nutritional and medicinal values such as nourishing liquid and lung, reinforcing the kidney, controlling nocturnal emissions and invigorating QI and YIN [2,3]. Yam polysaccharides are considered to be the main active components, including homopolysaccharides, heteropolysaccharides and glycoproteins, and its molecular weight ranges from thousands to tens of millions [4]. Existing studies have shown that yam polysaccharides possess a variety of biological activities, including anti-oxidation [5], immunomodulation [6,7], hypoglycemia [8] and regulating the intestinal flora [9,10]. These biological activities are influenced by their structure and degree of polymerization [2,11,12]. Zhao et al. [11] found that polysaccharide fractions with a higher uronic acid content (approximately 30%) and a smaller molecular weight (30–1000 kDa) had a higher antioxidant activity. Polysaccharide fractions with a higher content of monosaccharide compositions such as xylose, arabinose and galacturonic acid had better effects on anti-diabetic activity.

*S. thermophilus*, an oval, chain-shaped facultative anaerobic lactic acid bacteria, is often used along with *Lactobacillus bulgaricus* in the production of fermented milk to acidify curds and improve their texture characteristics [13]. *S. thermophilus* can also produce a variety of beneficial metabolites such as glycosidase, flavor compounds and extracellular polysaccharides during the fermentation process, thereby exerting multiple health effects on the body [14]. To ensure that *S. thermophilus*-related foods always maintain a high quantity of viable bacteria and biological activity during processing, storage, transportation and after being ingested by the body, a growing study has focused on identifying substances that can promote bacterial growth and reproduction, such as partial plant extract [15,16], flour [17], polysaccharide [18,19,20], polyphenol [21] and diosgenin [22].

To date, we have known that the connections between the bioactivities and structure characterizations of a polysaccharide are inseparable. However, how yam polysaccharides regulate the viable count and activity of lactic acid bacteria during fermentation, especially on the relevant structure–activity relationship and its mechanisms, is insufficiently understood. Herein, the growth-promoting effects of a yam polysaccharide on *S. thermophilus* were taken as a starting point to (1) preliminarily analyze growth-promoting mechanisms by comparing their molecular weight, monosaccharide compositions, and other basic structures and (2) further excavate and clarify the growth-promoting factors, and open new ways for intensively processing yam resources.

## 2. Materials and Methods

### 2.1. Materials

Fresh yam was produced in Huang-gang City (Hubei, China). The yam tuber was washed, peeled and sliced to 3 mm. After yam slices were dried using an RST-100RB heat pump dehumidification dryer (Shanghai Shiteng Electrical Co., Shanghai, China) at 55 °C for 10 h, it was ground and sieved through 100 meshes. Yam flour was stored in sealed packages in desiccators for the following experiments.

M17 broth was purchased from Hopebio Bio-Technology Co., Ltd. (Shandong, China); standards of dextrans, fucose, rhamnose, arabinose, galactose, glucose, etc., were all purchased from Sigma Chemical Co. (St. Louis, MO, USA). DEAE-cellulose 52 was purchased from Shanghai Yuanye Bio-technology Co., Ltd. (Shanghai, China). Choline chloride, urea, ethanol and other analytical reagents were from Sinopharm Chemical Reagent Co., Ltd. (Shanghai, China).

### 2.2. Strain and Culture Condition

*S. thermophilus* strain was from the strain resource library of our research group and stored at −80 °C. M17 broth was composed as follows (g/L): soybean peptone (5), peptone (2.5), casein peptone (2.5), yeast extract (2.5), beef extract (5), lactose (5), sodium ascorbate (0.5), β-glycerophosphate sodium (19) and magnesium sulfate (0.25), pH adjusted to (7.2 ± 0.2).

### 2.3. Extraction of Crude Yam Polysaccharide (CYP)

CYP was extracted using a DESs method [23,24], which can improve the yield of polysaccharide, while shortening the extraction time and reducing solvent loss. In brief, 1.00 g of dried yam flour was dissolved in 40 mL of DESs (choline chloride:urea = 1:5, molar ratio). After ultrasonic for 30 min and water bath processing at 90 °C for 40 min, the solution was enzymatically hydrolyzed with 150 U of amylase at 50 °C for 1 h and 1000 U of glucoamylase at 55 °C for 1 h. Then, the mixture was dealt with absolute ethanol overnight at 4 °C to obtain the precipitate after being centrifuged at 3000 rpm for 10 min. The precipitations were washed with absolute ethanol and 80% ethanol twice and re-dissolved in warm water. Then, Sevag reagent (CHCl_3_:CH_3_(CH_2_)_3_OH = 4:1) was added into the solution and fully oscillated until no visible protein layer could be seen. The polysaccharide solutions dealt with four volumes of absolute ethanol for 4 h, and centrifuged at 3000 rpm for 5 min. After dialysis, the CYP was lyophilized and stored for further investigation.

### 2.4. Separation of Yam Polysaccharide

CYP were separated as previously reported with slight modification [25,26]. Briefly, 100 mg of CYP was dissolved in 5 mL of deionized water and centrifuged at 12,000 rpm for 10 min. The supernatant was slowly loaded into a DEAE-cellulose 52 chromatographic column (1.6 cm × 70 cm), and was eluded using a gradient elution with a 0–1.0 mol/L NaCl solution at a flow rate of 1 mL/min. The eluant was fractionally collected at 5 mL/tube and monitored for total carbohydrate at 490 nm using a phenol-sulfuric acid method.

### 2.5. Growth-Promoting Effects of Yam Polysaccharide on S. thermophilus

Inoculum (1 mL) containing 7.0 lg CFU/mL of activated *S. thermophilus* was inoculated in M17 broth supplemented with CYP and four fractions to substitute lactose as the carbon source at 0.1% (*w*/*v*). After inoculation at 42 °C for 24 h, the number of colony-forming units (CFU) was enumerated, and the density was expressed as CFU/mL of medium. M17 broth was employed as the control group to compare the growth-promoting effects of yam polysaccharide.

### 2.6. Determination of Molecular Weight

The molecular weight of yam polysaccharide fractions was determined using high performance gel permeation chromatography (HPGPC). The polysaccharide fractions and dextran standards (1152, 5000, 11,600, 23,800, 48,600, 80,900, 148,000, 273,000, 409,800 and 667,800 Da) were accurately weighed and prepared as 5.00 mg/mL solutions, respectively. After being centrifuged at 12,000 rpm for 10 min, the supernatants were filtered with 0.22-micrometer polyethersulfone membranes. After that, 20 µL of the supernatants were analyzed on a LC-10A high performance liquid chromatographer (Shimadzu Instruments Co., Ltd., Kyoto, Japan) consisted of a BRT105-104-102 tandem gel column (300 mm × 8 mm) and a RI-10A differential refractive index detector. The mobile phase was 0.05 mmol/L NaCl at a flow rate of 0.60 mL/min, and the column temperature was kept at 40 °C.

### 2.7. Determination of Monosaccharide Compositions

Yam polysaccharide fractions (10.00 mg) were hydrolyzed with 10 mL of 3 M trifluoroacetic acid (TFA) at 120 °C for 3 h. After hydrolysis, excess TFA was removed by evaporating using a nitrogen blower and re-dissolved in 5 mL of deionized water. After that, the water solution (100 μL) was mixed with 900 μL of deionized water, and centrifuged at 12,000 rpm for 5 min. Then, 5 μL of the supernatant was analyzed using an ICS-5000 ion chromatographer (Thermo Fisher Technology Co., Ltd., Waltham, MA, USA) equipped with a Dionex CarbopacTMPA20 chromatographic column (150 mm × 3 mm) and a DC-2 electrochemical detector. The mobile phase: A was deionized water, B was a 15 mM NaOH solution and C was a 15 mM NaOH and 100 mM NaOAc solution; the elution gradients are detailed in Table 1. The rate of flow was 0.30 mL/min, and the column temperature was 30 °C.

### 2.8. Scanning Electron Microscope (SEM) Analysis

The yam polysaccharide fractions were adhered to the sample stage. After being coated by spraying gold, the samples were visualized using a JSM-6380LV SEM (JEOL Co., Ltd., Tokyo, Japan) under an energy of 20 kV.

### 2.9. Fourier Transform-Infrared Spectral (FT-IR) Analysis

The yam polysaccharide fractions were mixed with dry potassium bromide (KBr). After being ground in an agate mortar, the samples were pressed into slices under the action of a tableting machine and examined using a Nicolet iS5 FT-IR spectrometer (Thermo Fisher Technology Co., Ltd., Waltham, MA, USA) in the range of 4000–400 cm^−1^.

### 2.10. Nuclear Magnetic Resonance (NMR) Analysis

A 60 mg/mL solution of polysaccharide fractions was prepared with D_2_O. The ^1^H, ^13^C and ^135^DEPT-NMR spectrum of yam polysaccharide fractions were analyzed using an AV-400 NMR spectrometer (Bruck, Priyanka City, Germany) at 25 °C.

### 2.11. Statistical Analysis

All trials were carried out in triplicate and the data were analyzed by Origin 2019b and SPSS 19 and statistical significance was determined using Duncan’s multiple range test (*p* ≤ 0.05). NMR detection results were analyzed using MestReNova 14.

## 3. Results

### 3.1. Separation of Yam Polysaccharide

**Figure 1:**
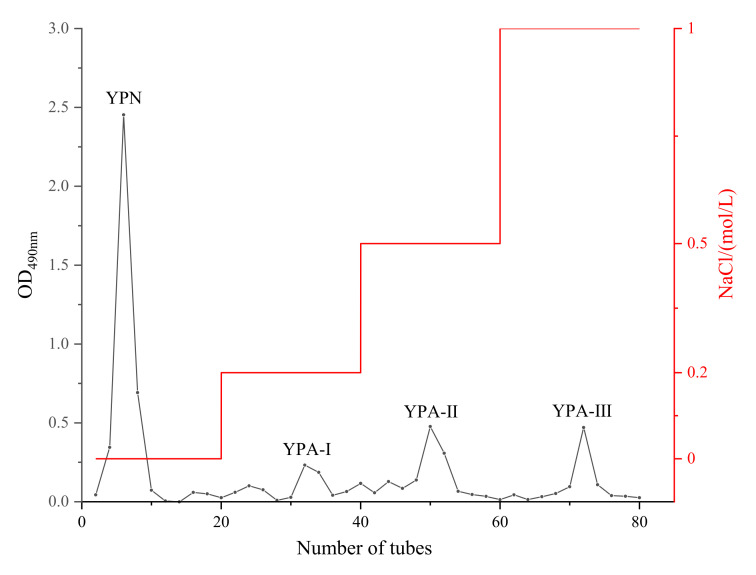
DEAE-cellulose 52 elution curve of CYP.

CYP was extracted using the DESs method with a yield of 11.3%. Due to the different charged nature of polysaccharide fractions, mixed polysaccharides are sequentially eluted from the ion exchanger by enhancing the ionic strength of the elution solvent (NaCl). DEAE-cellulose 52 is a commonly used medium for the separation of polysaccharides and is suitable for the separation of various neutral and acidic polysaccharides. CYP was separated after employing DEAE-cellulose 52; four eluting peaks were observed for one neutral yam polysaccharide and three acidic yam polysaccharides (Figure 1.) and designated as YPN, YPA-I, YPA-II and YPA-III. The four fractions were collected, concentrated, desalted and lyophilized for further experiments with yields of 20.7%, 1.4%, 11.2% and 3.6%, respectively, relative to the weight introduced by the separation method.

### 3.2. Effect of Yam Polysaccharide Fractions on the Growth of S. thermophilus

**Figure 2:**
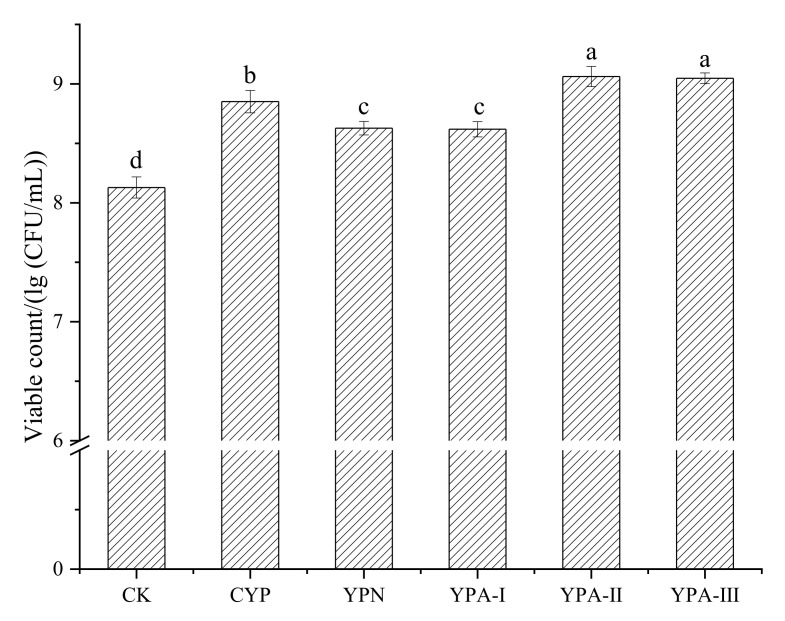
Growth-promoting effects of yam polysaccharide fractions on *S. thermophilus*. The same letters show the homogeneous groups.

From Figure 2, CYP and four fractions can be used as well as carbon sources for fermentation and can significantly promote the growth of *S. thermophilus*. When adding the same amount of polysaccharide, the number of viable bacteria in the YPA-Ⅱ group and YPA-Ⅲ group is roughly the same, with 9.062 lg (CFU/mL) and 9.047 lg (CFU/mL), respectively; but is significantly higher than that of the YPN group (8.628 lg (CFU/mL)) and YPA-I group (8.619 lg (CFU/mL)). Meanwhile, compared with the CYP group (8.851 lg (CFU/mL)), the viable counts in the YPN group and YPA-I group are also lower, indicating that the growth-promoting effects of YPN and YPA-I are relatively weak. The fermentation effects of lactic acid bacteria utilizing a polysaccharide depend on the enzymatic system of bacteria and also a polysaccharide’s structure characterization and its degree of polymerization [27], inferring that the main reason why yam polysaccharide fractions possess different growth-promoting effects relies on the differences in its structure and polymerization degree. Comprehensively considering their yields and growth-promoting effects, YPN, YPA-Ⅱ and YPA-Ⅲ are selected to explore their basic structure characterization in-depth.

### 3.3. Analysis of Molecular Weight

**Figure 3:**
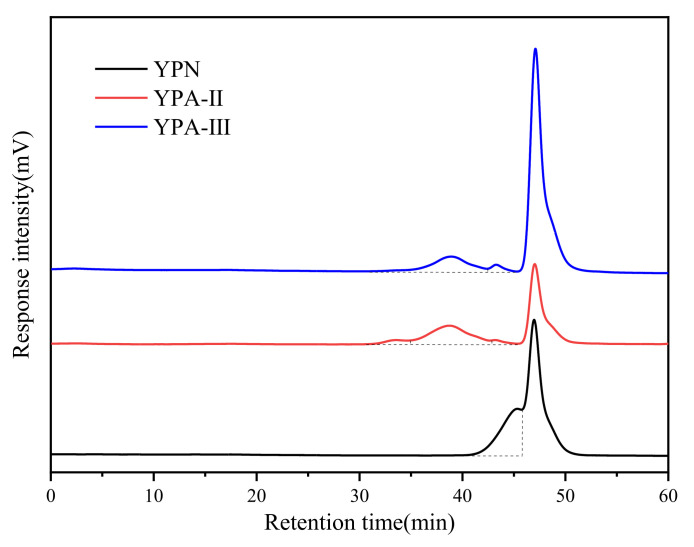
HPGPC profiles of yam polysaccharide fractions. The peak near 46.9 min is attributed to the mobile phase (0.05 mmol/L NaCl).

As can be seen from Figure 3 and Table 2, YPN showed a single absorption peak, indicating that YPN was a homopolysaccharide with a relatively uniform overall molecule distribution. The molecular weight (Mw) was 3209 Da according to the standard curve equation of dextran standards (lg *Mw* = 12.51242–0.19861*Rt*). YPA-Ⅱ has three absorption peaks (Mw: 733,506, 68,171, 8753 Da), and YPA-Ⅲ has two absorption peaks (Mw: 63,132, 8496 Da); also, comparative analysis revealed that YPA-Ⅱ and YPA-Ⅲ have two polysaccharide components with similar molecular weights (their retention time was near 38 and 43 min, respectively).

The biological activity of polysaccharides is closely related to the molecular weight, and only polysaccharide fractions in the appropriate molecular mass range of the same source have the best biological activity [28]. Considering that YPA-Ⅱ and YPA-Ⅲ have similar growth-promoting effects, their effects are better than YPN group, possibly because they have higher molecular weights, indicating that the polysaccharide fraction with a higher molecular weight may have a better growth-promoting effect in a certain range [28]. In addition, the composition of YPA-Ⅱ and YPA-Ⅲ is relatively complex, and the polysaccharide can be degraded into a variety of monosaccharide fractions after fermentation, which can provide more carbon species for *S. thermophilus* [29].

### 3.4. Analysis of Monosaccharide Compositions

**Figure 4:**
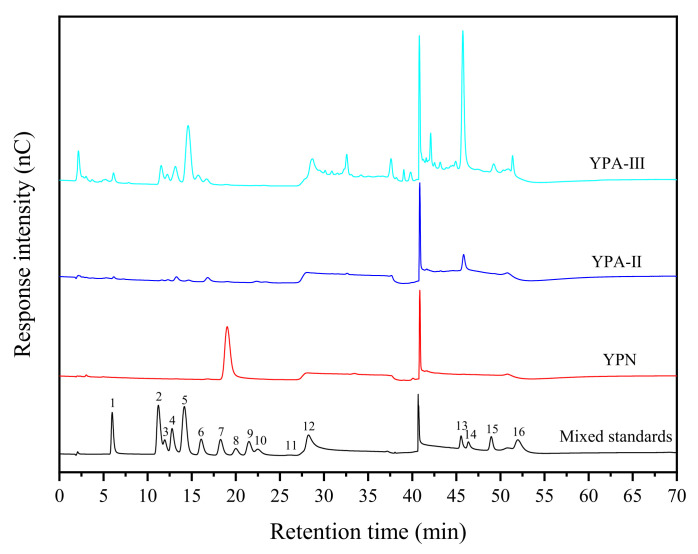
IC profiles of yam polysaccharide fractions. 1—Fucose, 2—Galactosamine hydrochloride, 3—Rhamnose, 4—Arabinose, 5—Glucosamine hydrochloride, 6—Galactose, 7—Glucose, 8—N-acetyl-D-Glucosamine, 9—Xylose, 10—Mannose, 11—Fructose, 12—Ribose, 13—Galacturonic acid, 14—Guluronic acid, 15—Glucuronic acid, 16—Mannuronic acid.

From the results presented in Figure 4 and Table 3, it is clear that three polysaccharide fractions were different in composition and the content of monosaccharides. YPN contains the following two monosaccharides: glucose and galactose, with a glucose content up to 99.2%. Thus, YPN can be approximately considered as a homopolysaccharide composed of glucose, consistent with the results of molecular weight analysis. Eight and nine monosaccharides were determined from YPA-Ⅱ and YPA-Ⅲ, respectively, of which seven monosaccharides are identical, and galacturonic acid was the most predominant component (48.5 and 66.1%, respectively). Zhu et al. [25] determined yam monosaccharide compositions using an HPLC precolumn derivatization PMP method but obtained different results. This discrepancy may be attributed to different polysaccharide extraction, separation and detection methods as well as yam sources and production places.

Monosaccharide compositions are one of the important factors affecting the growth-promoting effects of a polysaccharide, the composition of the polysaccharide unit determines the type of polysaccharide, and polysaccharides with different monosaccharide compositions as well as those with the same composition but different monosaccharide ratios exhibit different biological activities [30]. The growth-promoting effects of polysaccharide fractions is ranked in the order of YPA-Ⅱ ≈ YPA-Ⅲ > YPN > lactose (M17). YPA-Ⅱ and YPA-Ⅲ showed no significant difference in the growth-promoting effects on *S. thermophilus*, possibly because they contain a large amount of the same monosaccharides. YPA-Ⅱ and YPA-Ⅲ have relatively complex monosaccharide compositions and contain a higher galacturonic acid content, in which there may be some unique monosaccharides or high content of certain monosaccharide with high activity, which are more conducive to the growth of *S. thermophilus*. It is hypothesized that monosaccharides (such as arabinose, mannose, rhamnose, galactose and xylose) in YPA-Ⅱ and YPA-Ⅲ and their differences in content and structure will affect their growth-promoting effects to a certain extent, and the existence of galacturonic acid may give YPA-Ⅱ and YPA-Ⅲ better growth-promoting effects than glucose and lactose [29,30].

### 3.5. Analysis of SEM

**Figure 5:**
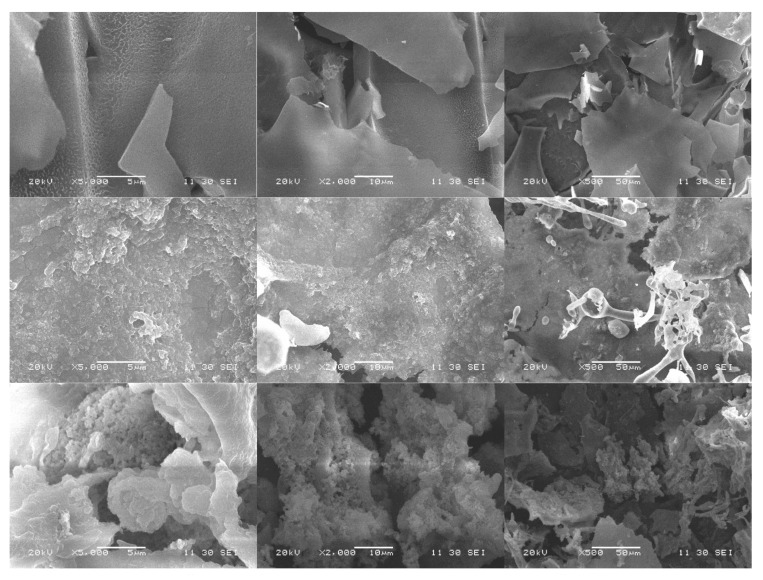
SEM images of yam polysaccharide fractions. The three lines of images from top to bottom represent the SEM results of YPN, YPA-II and YPA-III.

Figure 5 is a SEM image of yam polysaccharide fractions at a magnification of 5000, 2000 and 500 times. YPN has a large particle size and possesses a disorderly stacked sheet structure rather than spatial structure. YPA-Ⅱ appears to be irregular, clustered, or beaded in shape, similar to a porous sponge; YPA-Ⅲ has a similar appearance to YPA-Ⅱ, with rough and porous surfaces and a highly branched structure in some regions. The differences among YPN, YPA-II and YPA-III can be attributed to their monosaccharide compositions, especially the galacturonic acid content, and the configuration of their glycosidic bonds [31,32]. It was found that branched, spatially structured rapeseed polysaccharides exerted stronger growth-promoting effects than unbranched, planar rapeseed polysaccharides [33]. YPA-Ⅱ and YPA-Ⅲ have higher molecular weights and more branches and terminals, which are favorably utilized by lactic acid bacteria and are conducive to its hydrolyzation by glycosidase to a low molecular weight, thereby providing a sufficient carbon source for the fermentation system [34].

### 3.6. Analysis of FT-IR

**Figure 6:**
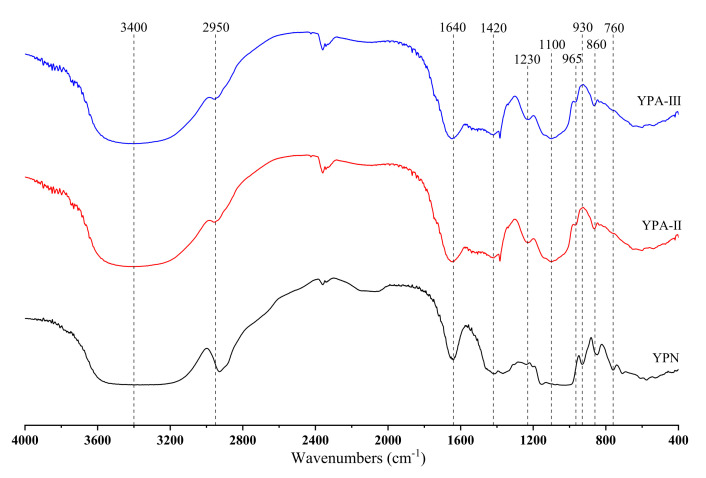
FT-IR spectrum of yam polysaccharide fractions.

The FT-IR spectrum of yam polysaccharide fractions (Figure 6) showed vibrational bands that are typical of carbohydrates. In the range of 400–4000 cm^−1^, the three fractions all have obvious absorption peaks near 3400 and 1640 cm^−1^ [35]. The strong broad absorption peak near 3400 cm^−1^ indicates the presence of inter- and intramolecular hydrogen bonds, which can be attributed to the superposition of multiple O-H stretching vibrations and may also contain N-H stretching vibrations (galactosamine hydrochloride and glucosamine hydrochloride in YPA-II and YPA-III). Comparing the spectrum of the three fractions found that YPN has stronger absorption peaks near 2930 and 1640 cm^−1^, indicating that YPN has higher contents of C-H bonds and crystal water than YPA-II and YPA-III [33]. In addition, YPN has no absorption peak near 1230 and 1100 cm^−1^, indicating that YPN does not contain an acetyl C-O bond and a C-O-C bond, indicating that YPN is a neutral polysaccharide. The absorption peaks near 930 and 760 cm^−1^ indicate that YPN has asymmetric stretching vibrations and symmetric stretching vibrations of the C-O-C skeleton of the D-glucopyranose ring [25]. The absorption peak near 860 cm^−1^ indicates that YPN has C-H variable-angle vibrations of α-glycosidic bonds [36]. YPA-Ⅱ and YPA-Ⅲ have absorption peaks near 960 cm^−1^, which are attributed to the rolling vibration of the methine group at the end of the pyran ring; it could be explained that rhamnose being present in YPA-Ⅱ and YPA-Ⅲ, which is consistent with the result of the monosaccharide compositions [28].

### 3.7. Analysis of NMR

^1^H-NMR is mainly used to resolve the glycosidic bond conformation of polysaccharides, and usually α-glycoside anomeric hydrogen exceeds 5.0 ppm, while the opposite is true for β-glycosides. The ^13^C-NMR spectrum can determine the number of anomeric carbons, the proportion of sugar residues and the sugar ring conformation, and most of the signals of anomeric carbons appear between 90–110 ppm, with α-type glycosides generally located at δ 90–100 ppm and β-type glycosides at 100–110 ppm. ^135^DEPT-NMR spectra can further determine the ^13^C NMR spectrum of the individual carbons (C, CH, CH_2_ and CH_3_), with a negative signal peak for seco-carbon at a pulse dump angle of θ = 135° [5].

**Figure 7:**
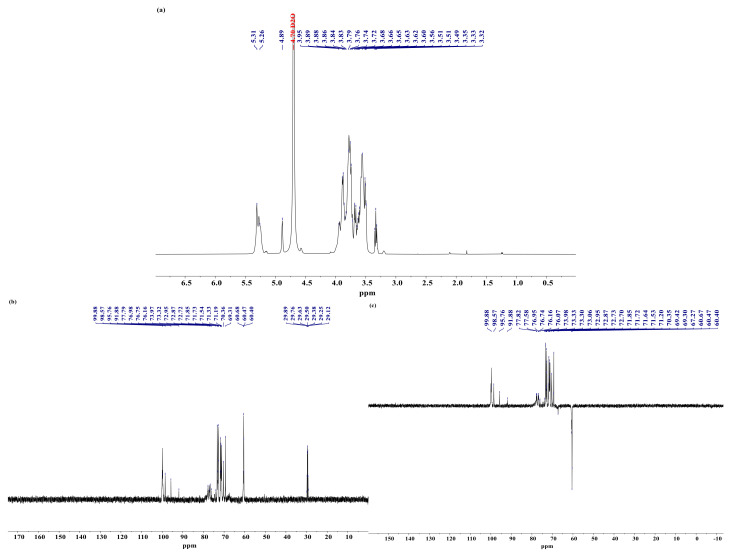
1D-NMR spectrum of YPN. (**a**) ^1^H-NMR; (**b**) ^13^C-NMR; (**c**) ^135^DEPT-NMR.

The ^1^H-NMR spectrum of YPN is shown in Figure 7a; the signal peaks at 5.31 and 5.26 ppm in the anomeric region indicate that YPN has two different α-type glycoside configurations. Combining the results of monosaccharide compositions, it is inferred that they are α-glycosidic bonds of glucose and galactose [37]. The anomeric carbon signal region of the ^13^C-NMR spectrum (Figure 7b) at 91.88–99.88 ppm confirmed this conclusion, which was consistent with the ^1^H-NMR spectrum. YPN has no obvious signal peak near 82 and 170 ppm, indicating no furanose and acetyl group. All these peaks further confirm that YPN is a neutral polysaccharide. In addition, YPN also has carbon signals between 60.51 and 77.82 ppm, indicating no hydroxyl substitution. Moreover, the ^135^DEPT-NMR spectrum was used to distinguish the primary carbon, secondary carbon, tertiary carbon and quaternary carbon. In Figure 7c, the negative signal peaks in the range of 60.40–67.27 ppm proved the presence of -CH_2_- (C5 and C6) [38].

**Figure 8:**
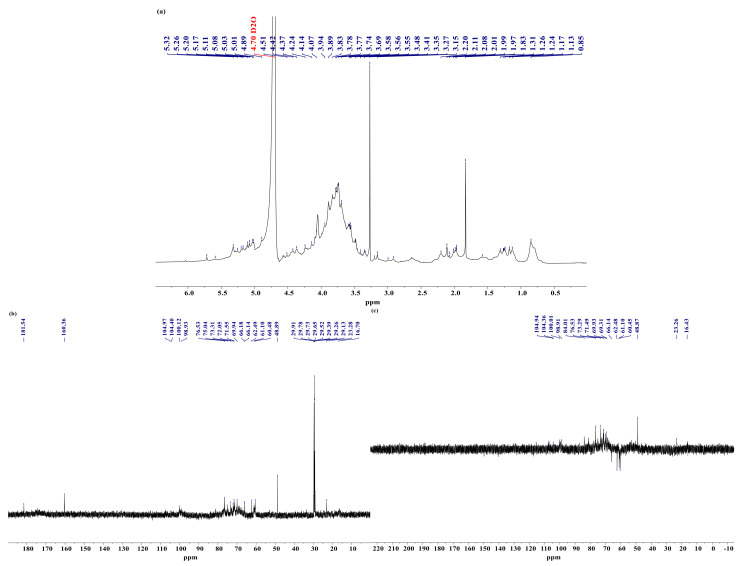
1D-NMR spectrum of YPA-II. (**a**) ^1^H-NMR; (**b**) ^13^C-NMR; (**c**) ^135^DEPT-NMR.

As shown in Figure 8a, YPA-Ⅱ has signal peaks on the left and right sides of 5.0 ppm. Among them, the signal peaks exceeding 5.0 ppm are densely arranged, indicating that YPA-Ⅱ contains a large amount of α-glycoside and the signal peak at 4.89 ppm is attributed to β-mannose [39]. The methyl proton peak of the ethoxy group appears at 0.85 ppm. It can be seen from the ^13^C-NMR spectrum (Figure 8b) that the signal peaks at 104.33, 100.14 and 98.96 ppm indicate that YPA-II contains both α-glycosides and β-glycosides. As YPA-Ⅱ has a more complicated monosaccharide composition, it may possess a ketose structure and more glycosidic bonds, which lead to a weaker peak signal in the anomeric carbon signal region. Moreover, the appearance of resonance signals at 81.64 and 83.82 ppm indicates that YPA-Ⅱ may contain furanose residues or the hydroxyl substitution of α-pyranose [25]. The carbonyl carbon signal at 174.16 ppm and the methyl signal at 23.28 ppm indicate the presence of acetyl groups in YPA-II, confirming that YPA-II is an acidic polysaccharide. The signal peak at δ16.59 ppm is attributed to the presence of rhamnose C6 [40]. Taking the ^135^DEPT-NMR spectrum (Figure 8c) into consideration, the appearance of negative signal peaks in the range of 60.43–66.14 ppm indicates the presence of methylene groups in YPA-II [5].

### 3.8. Analysis of Growth-Promoting Mechanisms

By and large, the growth-promoting mechanisms of *S. thermophilus* stimulated by yam polysaccharides could be explained as follows. First, polysaccharides with a higher molecular weight exert better growth-promoting effects. The growth-promoting effects of YPA-Ⅱ and YPA-Ⅲ was relatively preferable to that of YPN, presumably because their molecular weights were larger than that of the later. Second, monosaccharide compositions also influence the growth-promoting effects. YPA-Ⅱ and YPA-Ⅲ contained many monosaccharides, with the highest content of galacturonic acid; therefore, they could favorably promote the growth of *S. thermophilus*. Additionally, the different molar ratio of monosaccharides might also be attributed to their different growth-promoting effects. Third, the branch degree and complex spatial structure influences growth-promoting effects too. The growth-promoting effects of YPA-Ⅱ and YPA-Ⅲ were more significant than that of YPN and this phenomenon was presumably attributed to their spatial and branched structures. Fourth, the glycosidic bond conformations are equally important. In YPA-Ⅱ and YPA-Ⅲ, the existence of β-glycosides would make them have a better growth-promoting effect on *S. thermophilus*. This result is consistent with the previous finding that barley β-glucan can better promote the growth of *Lactococcus acidophilus*, which can be attributed to the fact that α-glycosidic bonds are easily hydrolyzed by α-glycosidases resulting in reduced activity, while β-glycosidic bonds facilitate coiling to form complex helical structures with better biological activity [41,42,43]. Beyond the above points, there might have been other mechanisms that need to be explored in-depth in the future.

## 4. Conclusions

In summary, the findings presented in this paper demonstrate that yam polysaccharides can significantly promote the growth of *S. thermophilus*, and the growth-promoting effects of different polysaccharide fractions are different. The structure–activity relationship analysis shows that the growth-promoting effects of yam polysaccharide fractions might be attributed to the differences in their molecular weight, monosaccharide compositions, microscopic forms and glycosidic bond configuration. The high growth-promoting activity of the yam polysaccharide may be more dependent on the higher molecular weights, the higher galacturonic acid content and the complex spatial structure, and the existence of β-glycosides would make the yam polysaccharide have a better growth-promoting effect on *S. thermophilus*. Overall, our investigations primarily analyze the growth-promoting mechanisms of yam polysaccharides and lay a foundation for the further development and utilization of yam polysaccharides. To understand the mechanisms deeper, it is necessary to further separate and purify yam polysaccharides and study its advanced structure and functions in detail.

## Figures and Tables

**Table 1:** Gradient elution procedure.

Retention Time/Min	Mobile Phase (%)		
	A	B	C
0.00	98.8	1.2	0.0
20.0	98.8	1.2	0.0
20.1	50.0	50.0	0.0
30.0	50.0	50.0	0.0
30.1	0.0	0.0	100.0
46.0	0.0	0.0	100.0
46.1	0.0	100.0	0.0
50.0	0.0	100.0	0.0
50.1	98.8	1.2	0.0
80.0	98.8	1.2	0.0

**Table 2:** Molecular weight data analysis of yam polysaccharide fractions.

Polysaccharide Fractions	Retention Time (min)	Mw/Da	Mn/Da	Mw/Mn
YPN	45.353	3209	2646	1.213
YPA-Ⅱ	33.469	733,506	405,556	1.809
	38.667	68,171	44,889	1.519
	43.158	8753	6703	1.306
YPA-Ⅲ	38.835	63,132	41,806	1.510
	43.223	8496	6521	1.303

Note: Mw, the weight average molecular mass; Mn, the number average molecular mass; Mw/Mn, the polydispersity index.

**Table 3:** IC data analysis of yam polysaccharide fractions.

Monosaccharides	Retention Time/Min	Molar Ratio		
		YPN	YPA-Ⅱ	YPA-Ⅲ
Fucose	5.984	ND	1.8	1.7
Galactosamine hydrochloride	11.217	ND	0.3	2.3
Rhamnose	11.942	ND	2.5	6.7
Arabinose	12.767	ND	7.6	8.5
Glucosamine hydrochloride	14.167	ND	0.6	8.6
Galactose	16.075	0.8	12.4	1.5
Glucose	18.275	99.2	ND	ND
Xylose	21.484	ND	11.0	ND
Mannose	22.484	ND	15.4	ND
Galacturonic acid	45.534	ND	48.5	66.1
Glucuronic acid	48.959	ND	ND	4.6

ND means that the monosaccharide was not detected in specific polysaccharide fractions.
